# Visibility forecast in Jiangsu province based on the GCN-GRU model

**DOI:** 10.1038/s41598-024-61572-8

**Published:** 2024-06-01

**Authors:** Huansang Chen, Yihang Xu, Zhiqiu Gao, Jia Kang, Yuncong Jiang, Zheng Li, Huan Shen

**Affiliations:** 1 School of Atmospheric Physics, Nanjing University of Information Science and Technology, Nanjing, 210044 China; 2 School of Computer Science and Technology, Hangzhou Dianzi University, Hangzhou, 310018 China

**Keywords:** Atmospheric science, Computer science

## Abstract

Low visibility weather easily leads to traffic accidents, posing threats to human life and property. To accurately forecast visibility, we conduct an empirical study focusing on Jiangsu Province. Firstly, we collect the monitoring data from meteorological stations and environmental stations for 2017-2018. Secondly, we analyze the changes in visibility from both spatial and temporal perspectives. Next, the maximum Relevance Minimum Redundancy (mRMR) algorithm is employed to select factors affecting visibility, finding that humidity and $PM_{2.5}$ concentrations are the primary factors. Finally, we propose GCN-GRU (Graph Convolutional Network and Gated Recurrent Unit) model for short-term visibility forecasting, which employs GCN to capture the interactions between stations and uses GRU to learn the interactions between times. Experimental results indicate that GCN-GRU outperforms the standalone GRU model and three machine learning models regarding 6-hour visibility forecasting. Compared to the best competitor, GCN-GRU achieves an average increase of 3.32% in Correlation Coefficient (CORR), a decrease of 17.52% in Root Mean Square Error (RMSE), a reduction of 26.62% in Mean Absolute Percentage Error (MAPE), and a decline of 16.53% in Mean Absolute Error (MAE).

## Introduction

Visibility reflects the transparency of the atmosphere. In meteorology, visibility is defined as the maximum horizontal distance at which an object (black and of moderate size) can be seen and identified against the sky background by a person with normal vision under prevailing weather conditions^1–3^. Low visibility poses a significant meteorological hazard, and its consequences, including economic losses, injuries, respiratory diseases, and fatalities, should not be underestimated.

Covering an area of 107,200 square kilometers, Jiangsu Province has a permanent population of 85 million. Economically, Jiangsu is one of China’s most competitive provinces, boasting the country’s largest manufacturing cluster. In recent years, due to rapid economic development and accelerated urbanization, the air quality in Jiangsu has gradually deteriorated. The frequency of low-visibility weather events has increased, severely impacting transportation, industrial production, and hindering economic growth. Traffic accident reports show that approximately 19.5% of traffic accidents occur during low visibility conditions such as heavy rain, snow, or fog^4^. Furthermore, numerous epidemiological studies have indicated that respiratory disease incidence and mortality rates significantly increase during low visibility conditions^5^.

Hence, it is imperative to uncover the spatio-temporal characteristics of visibility within Jiangsu Province, elucidate the key factors influencing visibility changes, and develop an atmospheric visibility forecasting model using machine learning algorithms based on various observation data and fundamental geographic information. Such efforts are critical for environmental protection, public health, and economic development.

In recent years, various methods have been proposed to forecast atmospheric visibility, mainly divided into numerical modeling forecasts and statistical modeling forecasts. Numerical modeling forecasts rely on established parametrization schemes based on physical and chemical mechanisms, such as the approach used by Goswami et al.^6^, which applied a dynamic simulation model to predict visibility changes induced by fog in Delhi. However, these forecasts largely depend on the accuracy of meteorological factors within specific processes, like dense fog, which limits their applicability. Moreover, the accuracy of the initial values in numerical predictions often gets affected by observational processes, background information errors, and potential issues related to incomplete descriptions of physical and dynamical processes^5^, leading to discrepancies between the forecasted results and actual meteorological conditions.

In contrast to numerical models, statistical models have less stringent requirements for input data accuracy. They can demonstrate better short-term predictive performance, especially when chemical processes and properties are not fully understood^7^. Additionally, statistical models have advantages in independence, efficiency, speed, and low computational demands. Traditional statistical forecasting methods establish models between visibility and meteorological elements based on empirical and linear models^8^, such as using air quality numerical models to build regional environmental monitoring forecasting systems^9^. However, visibility changes are a continuous and complex nonlinear process, so these models cannot fully describe the relationships between dependent and independent variables.

With the advancement of machine learning, some researchers have started using machine learning models, such as Support Vector Machines (SVM) and Decision Tree. For instance, Dutta et al.^10^ predict winter visibility at Kolkata Airport using Artificial Neural Network (ANN) and Decision Tree. They select pollutants and meteorological factors as influencing factors for visibility prediction. However, the long-term dependencies in the input data are difficult to capture by these methods, and vanishing gradient problems often appear in their time series prediction. To effectively overcome these issues, architectures more suitable for time series prediction, like Long Short-Term Memory (LSTM)^11^ and Gated Recurrent Unit (GRU)^12^, have emerged. For example, Miao et al.^13^ propose a novel LSTM framework for short-term fog forecasting.

Single models may not always achieve high predictive accuracy in specific applications. In the machine learning domain, ensemble learning is commonly used to address accuracy issues. The idea is to combine multiple learners in a way that complements their strengths, which can result in better fitting performance and more minor errors, ultimately improving the overall prediction outcome. Some researchers have begun to apply ensemble learning concepts to visibility prediction. For instance, Dai et al.^14^ employ Random Forest to achieve more accurate predictions.

In the realm of machine learning models for visibility forecasting, most studies focus on modeling single observation stations. For instance, Xu et al.^15^ develop a CNN-LSTM model to forecast visibility at Chongqing Jiangbei Airport. However, the trend in visibility changes is not only temporally dependent but also influenced by spatial factors within a given region. To consider both spatial and temporal factors, a common practice is to input features from both the forecasting station and its surrounding stations into the model without differentiation. For example, Tong et al.^16^ integrate multi-station features into an LSTM-Adaboost model, which overlooks the positional relationships between stations.

To comprehensively consider the impact of spatiotemporal factors on visibility, this study proposes a novel approach to predict visibility in Jiangsu Province more accurately. Initially, we apply the maximum Relevance Minimum Redundancy (mRMR) algorithm to select meteorological and environmental factors that significantly influence visibility. Then, we introduce an innovative GCN-GRU model that integrates a Graph Convolutional Network (GCN) and Gated Recurrent Unit (GRU). This model combines the strengths of GCN in capturing spatial relationships between meteorological stations with the efficiency of GRU in processing time series data.

Our main contributions are as follows:

- Conducted a comprehensive analysis of data from meteorological and environmental stations in Jiangsu Province, establishing an extensive database encompassing atmospheric visibility datasets and their spatiotemporal distribution characteristics.
- Identified the key factors affecting visibility in Jiangsu Province through meticulous data processing and analysis.
- Developed an innovative GCN-GRU model for efficiently and accurately forecasting visibility in Jiangsu Province.
- Demonstrating through extensive experiments that our model surpasses other comparative methods in predictive accuracy.

## Research data and area

### Data and data preprocessing

**Figure 1:**
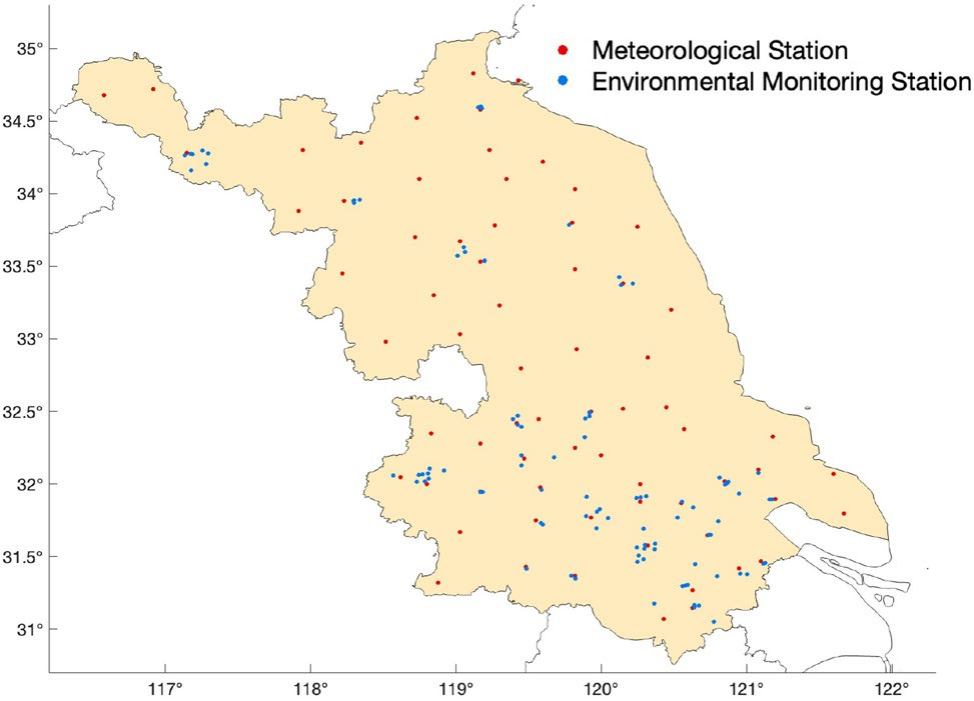
Spatial distribution of the 70 meteorological and 91 environmental monitoring stations in Jiangsu Province. The map was generated using Matlab R2023a (Matlab).

The data used in this study originates from the meteorological observation stations and environmental monitoring stations in Jiangsu Province from 2017 to 2018. The meteorological stations recorded 11 observation elements, namely hourly Temperature (TEM), Maximum Temperature (TEMmax), Minimum Temperature (TEMmin), Ground Surface Temperature (GST), Relative Humidity (RHU), Precipitation(PREC), Dew Point Temperature (DPT), Pressure (PRE), 2-meter Average Wind Direction (WD), 2-meter Average Wind Speed (WS) and Visibility (VIS). The environmental monitoring stations provided hourly concentration data for six atmospheric pollutants, namely $$PM_{2.5}$$, $$PM_{10}$$, $$ SO_{2}$$, $$ NO_{2} $$, $$ O_{3} $$ and *CO*. This study utilized historical data from 70 meteorological stations and 91 environmental monitoring stations. The spatial distribution of these stations is illustrated in Fig. 1.

Due to equipment malfunctions or other factors, observed data often contain missing and anomalous values. To monitor these anomalies effectively, this study employs the K-means clustering algorithm. K-means^17^ is a widely used clustering method that iteratively categorizes data points into several clusters, ensuring that points within each cluster are as similar as possible while being distinct from points in other clusters. In K-means, anomalies, often significantly different in characteristics from other data points, are likely to form distinct clusters. This feature enables us to identify anomalies by examining the size and density of these clusters, marking them as missing values. Unlike conventional approaches in most studies, such as direct deletion, interpolation, or substitution to handle missing values, considering the practical occurrence of data gaps, our study does not eliminate these missing values from the dataset. Instead, we annotate these missing values in the model. After training, the resulting model is capable of predicting visibility effectively, even in the presence of partial data gaps.

**Figure 2:**
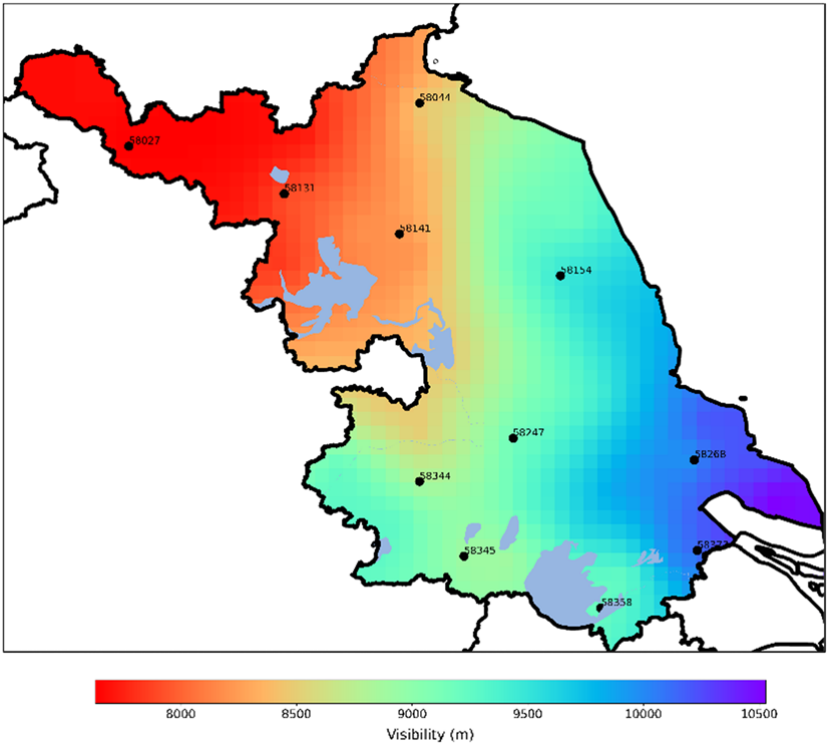
Annual average spatial distribution of visibility. The map was generated using Python 3.10 (Python) including matplotlib 3.7 and cartopy 0.21.

### Research area

Jiangsu Province lies in the Yangtze River Delta, on the eastern coast of mainland China, spanning longitudes $$116^\circ 21'\ $$E to $$121^\circ 56'\ $$E and latitudes $$30^\circ 45'\ $$N to $$35^\circ 08'\ $$N. Jiangsu borders the river and the sea, contains numerous lakes, and is characterized by its flat terrain, which consists of plains, water bodies, and low hills.

Due to spatial gaps in meteorological station data across the region, this study opted to use the ordinary Kriging interpolation method to enhance the accuracy of analyzing visibility distribution in Jiangsu Province. Kriging interpolation^18,19^, a spatial interpolation method based on statistical principles, accounts for the spatial relationship between sample points, providing reliable predictions at unobserved locations. The analysis results of visibility distribution are based on this interpolated data.

#### Annual average distribution characteristics

Figure 2 displays the annual average spatial visibility distribution for 2017 and 2018. In Jiangsu Province, the visibility is lower in the northwest and higher in the southeast. Coastal cities in the eastern part have an annual average visibility ranging between 7,000 and 9,000 meters. In contrast, the annual average visibility values in the western regions of Jiangsu Province are relatively lower, ranging between 5,000 and 7,000 meters. Notably, most areas of Xuzhou City have visibility less than 6,000 meters, showing a significant difference from other areas. This is primarily because in the northern parts of Jiangsu Province, especially in Xuzhou City, the production processes of heavy industries emit large amounts of pollutants, particulates, and dust. As a result, the air quality is subpar, leading to frequent smog occurrences, which reduces atmospheric visibility.

**Figure 3:**
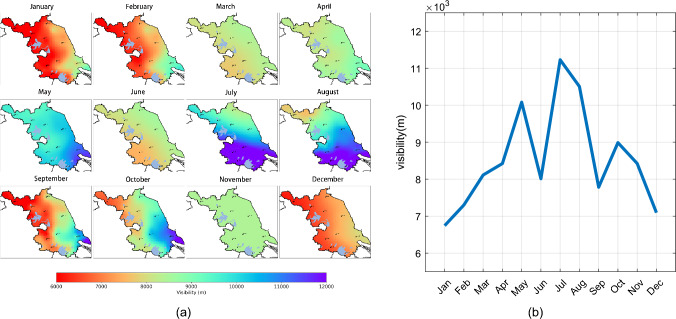
Monthly distribution of visibility in Jiangsu Province. The map **a** was generated using Python 3.10 (Python) including matplotlib 3.7 and cartopy 0.21.

#### Monthly distribution characteristics

Figure 3 illustrates the monthly visibility distribution in Jiangsu Province. As shown in Fig. 3a, January exhibits the lowest average visibility across all stations, with an average of 6,743 meters, while July boasts the highest average visibility at 11,229 meters. Generally, visibility tends to be lower during the autumn and winter, with peaks observed in May, July, and August.

The increased visibility in May, July, and August can be attributed to several factors. Firstly, the summer season witnesses frequent atmospheric convection, leading to quick vertical exchanges. Secondly, from late June to early July, the southern part of Jiangsu experiences the plum rain front, leading to frequent precipitation and high rainfall. This rainfall has a clean effect on atmospheric pollutants. Finally, during the summer, when the East Asian summer monsoon moves northward and encounters cold air from the north, it often results in mesoscale convective weather, which promotes the dispersion of pollutants and reduces atmospheric particulate matter.

Despite June being a summer month, its average visibility is relatively lower. This is primarily due to June being the most humid month in Jiangsu, creating favorable conditions for fog formation. Moreover, mid-to-late July and most of August are controlled by the subtropical high-pressure belt, with sunny and dry weather and low humidity. In contrast, winter in Jiangsu witnesses fewer precipitations, stable atmospheric layering, and poor diffusion conditions, leading to accumulated atmospheric pollutants and reduced visibility. Besides meteorological factors, coal-burning for heating in winter also significantly increases pollutants, leading to frequent smog and, hence, the lowest visibility values across all stations during the season.

#### Daily variation characteristics

**Figure 4:**
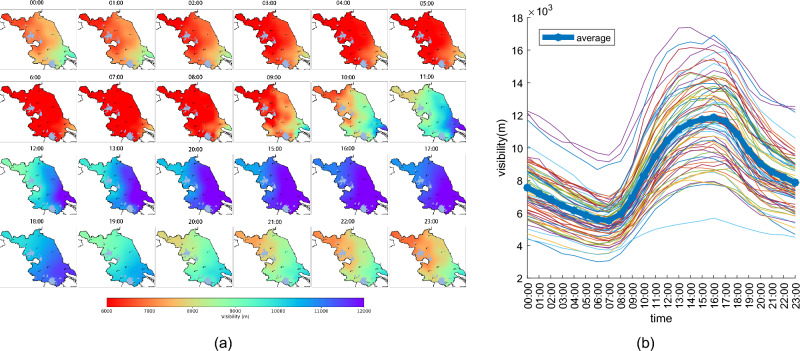
Average daily variation of visibility in Jiangsu Province. The map **a** was generated using Python 3.10 (Python) including matplotlib 3.7 and cartopy 0.21.

Figure 4 showcases the average daily variation in visibility across Jiangsu Province. As depicted in Fig. 4a visibility tends to be higher during the daytime and decreases at night. Throughout the day, the visibility follows a pattern of being lower in the northwest and higher in the southeast.

Figure 4b provides insights into the daily variation trends across all 70 stations and the average daily variation trend across all stations. There is a distinct daily variation trend in visibility across Jiangsu, with minimal variations between different regions. Visibility rises from 7:00 to 16:00, peaking at 16:00 with an average visibility of 11,861 meters across all stations. From 16:00 to 7:00 the next day, visibility declines, reaching its lowest at 7:00 with an average of 5,528 meters.

The daily variation in visibility follows a periodic pattern. It is at its lowest during the early morning hours until sunrise, a period characterized by higher relative humidity, slower wind speeds, and prevalent radiation cooling, all of which contribute to favorable conditions for fog formation. Additionally, the stable atmospheric boundary layer and inversion layer limit the transport and diffusion of aerosol particles, causing their accumulation near the bottom of the atmospheric boundary layer. This increases the likelihood of haze, with morning rush hour traffic exacerbating the situation.

Following sunrise, the ground absorbs short-wave solar radiation, leading to a rise in near-surface temperatures, a decrease in relative humidity, increased atmospheric boundary layer instability, the lifting and eventual disappearance of the inversion layer, heightened vertical transport, and gradual improvements in visibility. In the afternoon, turbulence mixing and thermal convection intensify further while ground wind speeds peak, accelerating the dispersion of fog and particulate matter in the air. These processes result in the highest visibility levels observed during the afternoon.

## Methodology

### mRMR Algorithm

To accurately identify and prioritize the meteorological and environmental factors that most significantly impact visibility, and to ensure that the most relevant predictors are included in the subsequent modeling phase, we utilized the mRMR algorithm to select the observation elements from the environmental and meteorological stations. The maximum Relevance Minimum Redundancy (mRMR)^20^ is a feature selection algorithm designed to select the most informative subset of features from high-dimensional data. The central idea behind this algorithm is to simultaneously maximize the relevance of features with the target variable while minimizing the redundancy among the features themselves.

In the mRMR algorithm, the first step involves calculating the relevance between each feature and the target variable. This relevance is typically measured using metrics like correlation coefficients or mutual information. Subsequently, the algorithm calculates the redundancy between pairs of features to ascertain their similarity or correlation. The objective function of mRMR is defined as:

1 $$\begin{aligned} \max \sum _{i} MI(X_i,Y) - \sum _{i,j} MI(X_i,X_j), \end{aligned}$$

where $MI(X_i,Y)$ represents the correlation information between feature $X_i$ and the target variable *Y*, and $MI(X_i,X_j)$ represents the redundancy information between feature $X_i$ and feature $X_j$. The mutual information is defined as:

2 $$\begin{aligned} MI(X,Y) = \sum _{x \in X} \sum _{y \in Y} p(x,y) \log \frac{p(x,y)}{p(x)p(y)}, \end{aligned}$$

where *p*(*x*, *y*) is the joint probability distribution of *X* and *Y*, *p*(*x*) and *p*(*y*) are the marginal probability distributions of *X* and *Y*, respectively.

By utilizing the abovementioned approach, we can select a set of features highly correlated with visibility and exhibit low redundancy amongst each other, thereby providing quality input features for subsequent modeling.

### GCN-GRU model

In machine learning models for visibility forecasting, most of the existing studies focus on single-station modeling. These studies typically treat the predicted station and a few surrounding stations equally, considering all the features from these stations as the input to the model.

    However, we argue that these approaches neglect the spatial interactions between meteorological factors across different stations. For example, at a given time, Station A observes warm and humid air mass and light westerly winds from Station B, located to its west. In the next time step, Station A observes the advection fog from Station B. A spatio-temporal-based model can easily model such scenarios and make accurate predictions in similar situations. On the other hand, traditional forecasting models, which lack spatial awareness in their input representation, struggle to make accurate predictions.

    Therefore, we propose a model named GCN-GRU (Graph Convolutional Network and Gated Recurrent Unit), where the GCN module focus on spatial features and the GRU module deals with temporal features. In terms of space, we represent the relationships between stations using a graph structure and utilize GCN to learn the spatial representation of these stations. In terms of time, we feed the learned spatial representation at each time step into the GRU network, allowing the model to learn the interaction between the spatial representations at different time steps.

**Figure 5:**
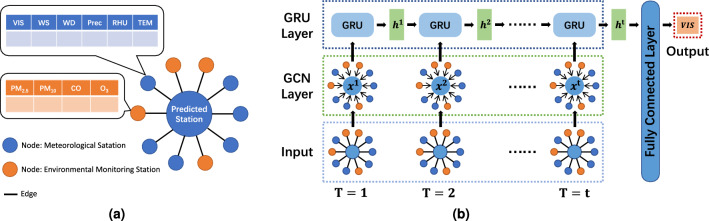
Model.

#### Graph structure

Firstly, we utilize the graph structure to represent the relationships between meteorological and environmental monitoring stations. Specifically, we represent the predicted station and its closest ten stations using the graph structure. Figure 5a illustrates the graph structure of the predicted station and its surrounding stations, where blue nodes represent meteorological stations and orange nodes represent environmental stations. Additionally, we use undirected edges to represent the relationships between the stations. The surrounding ten stations and the central predicted station are connected by edges, indicating a bidirectional relationship. We only consider the influence of the surrounding stations on the predicted station and do not account for interactions among the surrounding stations. Thus, no edges are constructed between the surrounding stations.

#### Graph convolution network layer

Secondly, we utilize Graph Convolution Network (GCN)^21^ to aggregate information from the surrounding stations onto the central predicted station. GCN is a deep learning technique designed explicitly for graph structures, extending the principles of traditional Convolutional Neural Networks (CNNs)^22^ to accommodate graph data. GCN aggregates information from neighboring nodes to the target node, and after a non-linear transformation, obtains the overall representation of the target node.

As shown in Fig. 5b, in the GCN layer, for the graph structure at time step $t$, we apply graph convolution operations to obtain the spatial representation $x^t$ of the predicted station.

#### Gated recurrent unit layer

Thirdly, we use Gated Recurrent Unit (GRU)^12^ to capture the interactions of spatial representation between different time steps. GRU is a variant of Recurrent Neural Network (RNN) for processing sequence data, aiming to address the vanishing gradient problem inherent in RNN while retaining the capability of RNN to capture long-term dependencies in sequences.

As shown in Fig. 5b, at time step 1, the spatial representation $x^1$ is fed into the GRU, resulting in the spatio-temporal representation $h^1$ for that time step. At time step 2, the previous spatio-temporal representation $h^1$ and the current spatial representation $x^2$ are both input into the GRU, yielding the spatio-temporal representation $h^2$ for that time step. This process is repeated until the GRU layer outputs the spatio-temporal representation $h^t$ for time step $t$.

#### Fully connected layer

Finally, we pass the spatio-temporal representation $h^t$ through a fully connected layer. The fully connected layer enables complex non-linear transformations and helps the model learn higher-level representations, thereby mapping the features to the output space.

## Experiment

### Evaluation metrics

In this study, we use Root Mean Square Error (RMSE), Mean Absolute Error (MAE), Mean Absolute Percentage Error (MAPE) , and Correlation Coefficient (CORR) as metrics to evaluate the performance of the models.

**RMSE** is a commonly used metric for evaluating the accuracy of a predictive model. It measures the differences between observed values and predicted values, with lower values indicating better accuracy. The formula for RMSE is defined as

3 $$\begin{aligned} \text {RMSE} = \sqrt{\frac{1}{n} \sum _{i=1}^{n} (y_i - \hat{y}_i)^2}, \end{aligned}$$

where $y_i$ is the observed value, $\hat{y}_i$ is the predicted value, and $n$ is the number of observations.

**MAE** is another metric used to assess the accuracy of a model’s predictions. Like RMSE, lower MAE values indicate better accuracy. The formula for MAE is

4 $$\begin{aligned} \text {MAE} = \frac{1}{n} \sum _{i=1}^{n} |y_i - \hat{y}_i|. \end{aligned}$$

**MAPE** measures the average percentage difference between observed values and predicted values, relative to observed values. MAPE is expressed as a percentage, with lower values indicating better accuracy. The formula for MAPE is

5 $$\begin{aligned} \text {MAPE} = \frac{100\%}{n} \sum _{i=1}^{n} \left| \frac{y_i - \hat{y}_i}{y_i} \right| . \end{aligned}$$

**CORR** is a statistical measure that quantifies the degree of association between two variables. The value of CORR usually ranges between -1 and 1. A value close to 1 indicates a strong positive correlation between the two variables, while a value close to -1 indicates a strong negative correlation. The formula for CORR is:

6 $$\begin{aligned} \text {CORR} = \frac{\sum _{i=1}^{n} (x_i - \bar{x})(y_i - \bar{y})}{\sqrt{\sum _{i=1}^{n} (x_i - \bar{x})^2 \sum _{i=1}^{n} (y_i - \bar{y})^2}}, \end{aligned}$$

where $x_i$ and $y_i$ are the individual data points, and $\bar{x}$ and $\bar{y}$ are the means of $x$ and $y$ respectively.

These metrics are essential to gauge the effectiveness and accuracy of the models in predicting visibility and help in understanding how closely the predictions align with the actual observations.

### Comparative models

To better evaluate the performance of GCN-GRU, we introduce four comparative models as follows:

**GRU**, a deep learning model, simplifies the design of LSTM^11^ by combining the forget gate and input gate into a single update gate. Additionally, it merges the cell state and the hidden state of LSTM, leading to a more architectural model with fewer parameters.

**Random Forest**^23^, a robust ensemble learning model, classifies and regresses by combining multiple decision trees. Its primary advantage is its ability to handle high-dimensional data with numerous features and its robustness to outliers and missing data. The core idea of the random forest is to construct multiple decision trees by randomly selecting training samples and feature subsets. Then, by averaging or majority voting on the predictions of these trees, it produces the final prediction. This method can reduce the overfitting problem and enhance the model’s generalization ability.

**XGBoost**^24^, a gradient boosting tree model, performs ensemble learning by progressively adding tree models within the gradient boosting framework. XGBoost has gained widespread attention for its outstanding predictive performance and computational efficiency. It optimizes the model by minimizing a loss function, using gradient information to update the tree model, thereby gradually reducing prediction errors. Furthermore, XGBoost introduces regularization to prevent overfitting.

**LightGBM**^25^, a lightweight ensemble learning model based on gradient boosting trees, adopts a histogram-based algorithm to improve computational efficiency. LightGBM proposes the Gradient-based One-Side Sampling (GOSS) to remove small-gradient instances and introduces Exclusive Feature Bundling (EFB) to reduce feature dimensions. These techniques substantially reduce memory consumption and computation time while maintaining good predictive performance.

As the time series models, both GRU and GCN-GRU can employ multi-step and single-step forecasting. Multi-step forecasting continuously generates predictions for multiple future time steps, suitable for long-term trend prediction but potentially accumulates initial prediction errors over time. In contrast, single-step forecasting predicts the value for only the next time step, quickly adapting to recent data changes and suitable for scenarios requiring rapid response. Therefore, a total of seven models were involved in the comparison: GCN-GRU (Single), GCN-GRU (Multi), GRU (Single), GRU (Multi), LightGBM, Random Forest, and XGBoost. Moreover, parameters for Random Forest, XGBoost, and LightGBM were optimized using Bayesian search to enhance the model’s generalizability. As for the time series models, we empirically set the optimal parameters.

### Analysis of visibility influencing factors

To accurately identify and analyze the key factors influencing visibility in Jiangsu Province, we employed the maximum Relevance Minimum Redundancy (mRMR) algorithm for feature selection. We identified several critical meteorological and environmental factors that significantly impact visibility.

**Figure 6:**
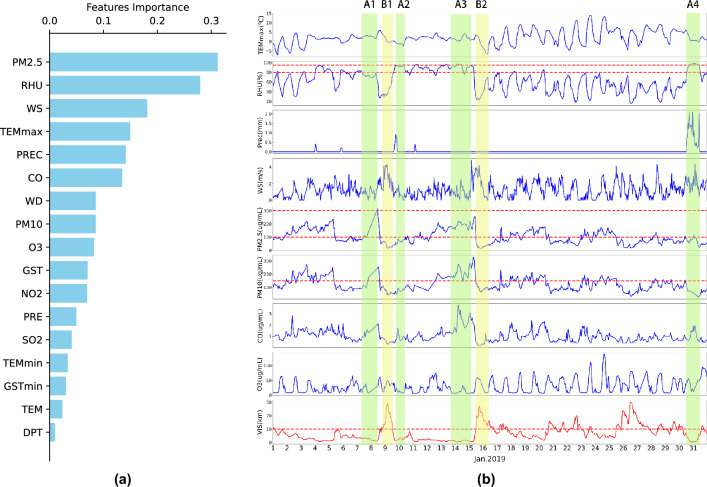
Impact factor screening.

From Fig. 6a, it can be inferred that Relative Humidity (RHU) and $PM_{2.5}$ concentration are the most significant factors affecting visibility. Besides, there is a certain redundancy between some variables, such as Temperature (TEM), hourly Maximum Temperature (TEMmax), and hourly Minimum Temperature (TEMmin). Only one among them needs to be selected for model training. Based on the ranking results of mRMR and previous research^26–28^, we choose Relative Humidity (RHU), 2-meter Wind Speed (WS), 2-meter Wind Direction (WD), Precipitation (PREC), hourly Highest Temperature (TEMmax), $PM_{2.5}$, $PM_{10}$, *CO* and $O_{3}$ concentration as the input variables of the model.

Figure 6b displays the variation of visibility and other elements for Station 58027 (Coordinates: $117.15^\circ$E, $34.28^\circ$N) in January 2019. The red dashed lines indicate threshold values for some elements. For instance, low visibility events occurring below 80% relative humidity are identified as haze, between 80% and 95% as fog-haze, and above 95% as fog. $PM_{2.5}$ concentration greater than 100 indicates air pollution and more than 300 indicates severe pollution. $PM_{10}$ concentration greater than 100 is also deemed as air pollution. Visibility below 10 kilometers is classified as a low-visibility event.

**Ax** represents periods of low visibility, while **Bx** represents periods of high visibility. For instance, during the **A1** period, the low visibility was caused by fog-haze, with air humidity ranging between 70% and 80%, low wind speed, and high concentrations of several pollutants. By the **B2** period, the haze had dissipated, primarily due to the gradual increase in wind speed. The **A2** period witnessed the occurrence of thick fog, caused by short-term rainfall that increased the air humidity and reduced the air pollutants due to the washing effect of the rain. The **A3** period represents a typical fog-haze scenario, with humidity levels between 80% and 95% and high concentrations of pollutants. During the **B2** period, due to reduced humidity and increased wind speed, the fog-haze dispersed, improving visibility. The low visibility during **A4** was caused by precipitation. Despite significant wind speeds, short-duration heavy rainfall can suddenly reduce visibility. Once the rainfall ceased, the visibility returned to normal.

    Following the analysis of visibility variations during periods marked as **Ax** (low visibility) and **Bx** (high visibility), we further employed the Spearman’s Rank Correlation Coefficient^29^ to assess the relationships between visibility and various meteorological and environmental factors. This method is particularly effective for non-normally distributed data and is less sensitive to outliers, revealing the rank-order relationships between variables. The analysis results are presented in Table 1. Our analysis indicates that factors such as relative humidity, $PM_{2.5}$ concentration, and *CO* concentration exhibit negative correlations with visibility, suggesting that an increase in these factors typically leads to a decrease in visibility. Conversely, the highest temperature, wind speed, and ozone concentration show positive correlations with visibility, implying that visibility may improve under these conditions.

**Table 1:** Spearman’s Rank Correlation Coefficients between Visibility and Various Factors.

Factor	Spearman’s Rank Correlation Coefficient
Relative Humidity	-0.60**
$PM_{2.5}$ Concentration	-0.59**
*CO* Concentration	-0.53**
$PM_{10}$ Concentration	-0.46**
Precipitation	-0.18**
Maximum Temperature	0.20**
Wind Speed	0.31**
$O_{3}$ Concentration	0.40**

** indicates that the values have passed the significance test

### Model forecasting

We train the model on data from year 2017 to 2018. The dataset was divided into 60% for training, 20% for validation, and 20% for testing to ensure comprehensive training and effective validation under various data conditions. Since historical information beyond 72 hours minimizes future visibility trends, a 72-hour time window was selected as a critical parameter for model training.

**Table 2:** Locations of Predicted Stations and Their Surrounding Meteorological and Environmental Stations.

Predicted Station	City	Surrounding Meteorological Stations	Surrounding Environmental Stations
58027	Xuzhou	58013, 58012, 58026, 58130, 58131	99452, 99451, 99454, 99453, 99457
58044	Lianyungang	58040, 58047, 58041, 58036, 58048, 58045, 58038, 58140, 58049	99237
58131	Suqian	58130, 58035, 58026, 58135, 58038, 58132, 58036, 58141	99343, 99342
58141	Huai’an	58145, 58140, 58132, 58139, 58038	99177, 99175, 99174, 99176, 99178
58154	Yancheng	58146, 58158, 58150, 58251, 58143, 58243, 58049	99458, 99460, 99459
58247	Zhenjiang	58249, 58246, 58244, 58252, 58341	99492, 99368, 99367, 99366, 99046
58345	Changzhou	58342, 58346, 58340	99787, 99788, 99755, 99896, 99756, 99895, 99493
58358	Suzhou	58359, 58349	99348, 99876, 99877, 99878, 99346, 99351, 99345, 99349
58377	Taicang	58356, 58352	99866, 99867, 99780, 99779, 99350, 99697, 99695, 99721
58268	Nantong	58360, 58259, 58264	99826, 99271, 99269, 99721, 99270, 99822, 99268
58344	Nanjing	58340, 58242, 58238, 58252	99769, 99770, 99257, 99490, 99255, 99258

    To validate the model’s applicability and robustness across different geographic locations, 11 meteorological stations within Jiangsu Province were selected for testing, considering the representativeness of geographical distribution and data completeness. Table 2 and Fig. 2 show the specific station information.

**Figure 7:**
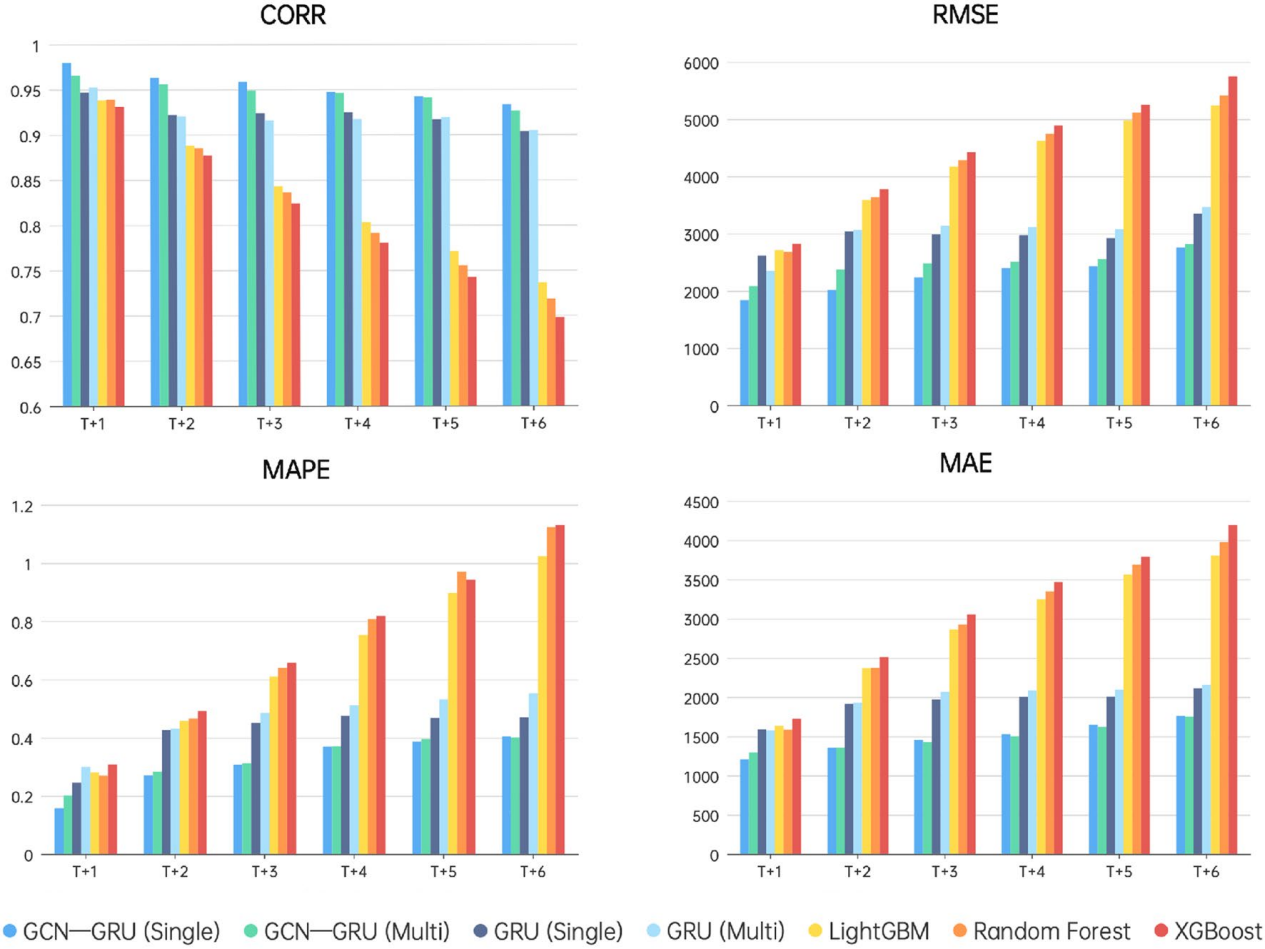
Comparison of Test Set Results for GCN-GRU Single Step Prediction, GCN-GRU Multi step Prediction, GRU Single Step Prediction, GRU Multi step Prediction, LightGBM, Random Forest and XGBoost.

Figure 7 illustrates the performance of seven models for forecast horizons of 1–6 h. As the forecast horizon increased, the correlation coefficient (CORR) decreased, while the root mean square error (RMSE), mean absolute percentage error (MAPE), and mean absolute error (MAE) increased, indicating a decline in forecasting accuracy over time. Compared to traditional machine learning models, time series prediction models significantly improved the accuracy of visibility forecasts, especially for more extended forecast periods.

Our analysis also found that single-step prediction results were almost identical to multi-step predictions but required substantially less time. Therefore, single-step prediction was chosen as the primary method for visibility forecasting. Additionally, when comparing the GRU-GCN model to the standalone GRU model, the GRU-GCN model exhibited improvements. In 6-hour forecasts, the average CORR increased by 3.32%, RMSE decreased by 17.52%, MAPE reduced by 26.62%, and MAE decreased by 16.53%.

### Case analysis: forecasting application

**Figure 8:**
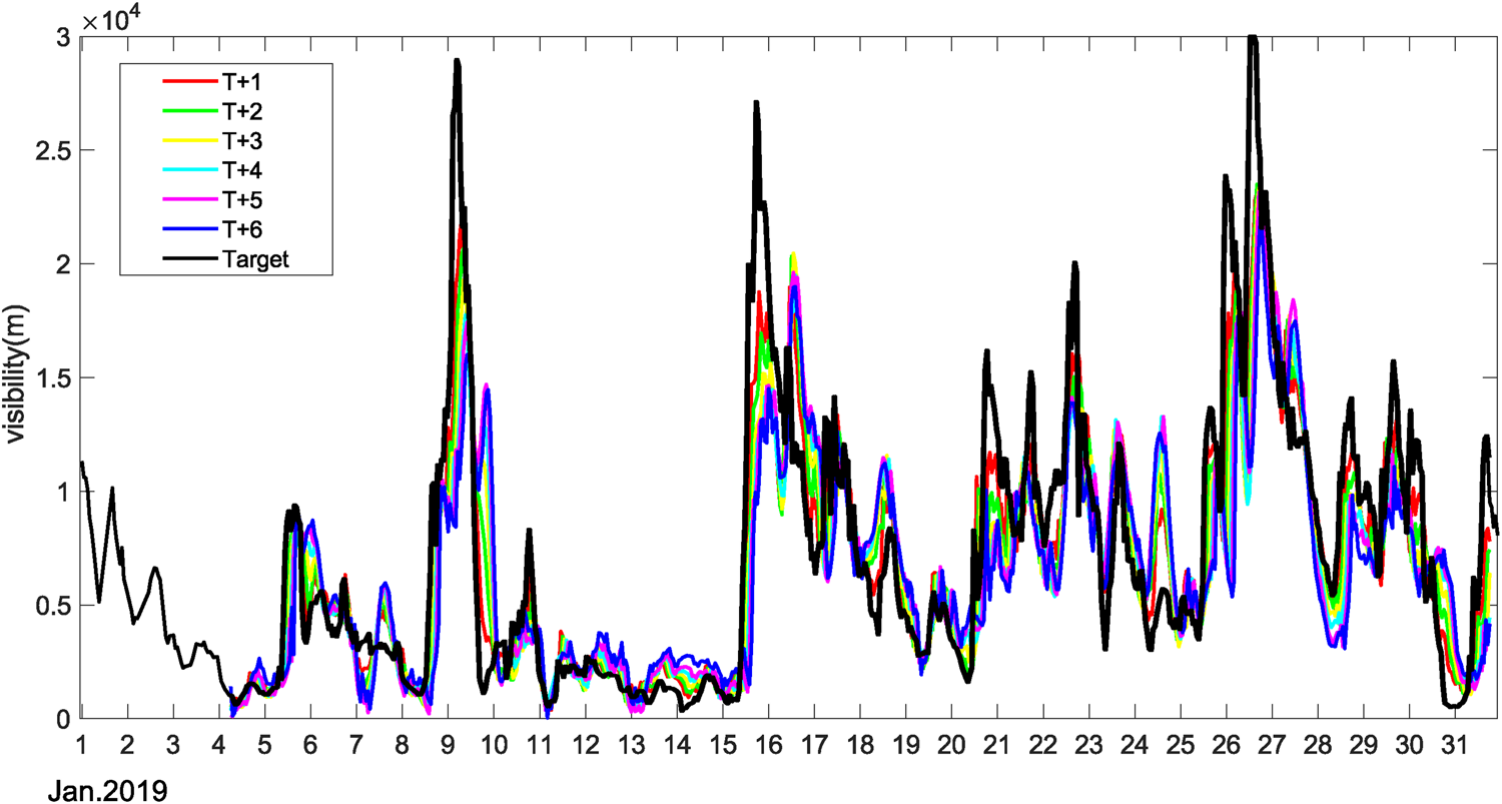
January 2019 GCN-GRU rolling forecast results.

In our study, particular attention was given to the performance of the GCN-GRU single-step prediction model in practical application scenarios. Taking Station 58027 as an example, we conducted rolling forecasts for data from January 2019, and the results are shown in Fig. 8. In this case, the initial 72 hours of data served as historical input and thus did not contain forecast values. The results showed that the trends predicted by the GCN-GRU model generally aligned with the actual values, but there were discrepancies in the prediction of extreme values. Specifically, the model tended to slightly overestimate at lower values and underestimate at extremely high values.

This consistency in trend prediction and deviation in extreme values could be attributed to several factors. Firstly, the distribution of training data might be sparse in the extreme value regions, making it challenging for the model to learn behaviors in these situations accurately. Secondly, the model’s structure might be more suited to capturing general trends rather than extreme events. Moreover, predicting extreme values in time series data is inherently more challenging, especially in dynamic and changing environmental conditions.

However, it is essential to note that such inaccuracies in extreme value prediction do not significantly impact the model’s effectiveness in practical applications. In most cases, particularly in visibility forecasting scenarios, it is more crucial to capture changes in future trends rather than to predict each specific value accurately. In this regard, the GCN-GRU model demonstrated good performance, providing valuable insights for timely and effective decision-making.

In conclusion, although the GCN-GRU model requires improvements in predicting extreme values, it still exhibits strong potential and practicality in real-world applications. Future work could focus on optimizing the model structure, improving data preprocessing strategies, and incorporating new algorithms and techniques to enhance the model’s predictive accuracy in various situations, especially for extreme values.

## Conclusion

Based on meteorological and environmental monitoring data from Jiangsu Province spanning years 2017 to 2018, this study investigates the spatio-temporal characteristics of atmospheric visibility within the region. It identifies critical meteorological and environmental factors that significantly influence visibility. By using GCN to learn spatial interactions between stations and GRU to learn temporal interactions between moments, this study has successfully developed a more accurate model for visibility prediction. The primary findings are summarized as follows:

- Atmospheric visibility in Jiangsu Province exhibits distinct spatio-temporal characteristics. Spatially, visibility generally increases from the northwest to the southeast, with slight variations in different months. Temporally, there are apparent daily and monthly cyclical changes, with the highest monthly averages often occurring in August and the lowest in January. Within a day, visibility first rises and then declines, typically reaching its lowest point at dawn and peaking in the afternoon.
- Through mRMR algorithm, nine influential yet relatively independent factors affecting visibility are selected: Relative Humidity, Wind Speed, Wind Direction, Precipitation, Maximum Temperature, $PM_{2.5}$, $PM_{10}$, *CO*, $O_{3}$ concentration. Visibility positively correlates with Wind Speed and negatively correlates with Relative Humidity, $PM_{2.5}$, $PM_{10}$, *CO*, and $O_{3}$ concentration.
- In the context of visibility prediction, the spatial relationships among stations are first incorporated into a graph structure. By leveraging graph convolution, information from surrounding stations is aggregated and subsequently input into the GRU for time series prediction. The resulting GCN-GRU model, compared to the standalone GRU model, demonstrates notable improvements in 6-hour visibility prediction. Specifically, it achieves an average CORR increase of 3.32%, a reduction in RMSE of 17.52%, a decrease in MAPE of 26.62%, and a decline in MAE of 16.53%.

## Limitations and suggestions

- This study only considers ground station elements when modeling. Upper-atmospheric elements (e.g., temperature inversion and boundary layer height) can also impact ground-level visibility changes. Future research can incorporate sounding data for a more comprehensive study.
- Due to computational resource constraints, the study models the distribution of stations by only considering the ten nearest stations. Suppose a broader range of stations (e.g., the entire Jiangsu Province) can be simultaneously input into a single graph structure. In that case, the model might better grasp the overall spatio-temporal distribution of visibility in Jiangsu Province.

## Data Availability

The dataset and code are available at https://github.com/Lucienxhh/GCN_GRU.
